# Protein hydrolysates enhance recovery from drought stress in tomato plants: phenomic and metabolomic insights

**DOI:** 10.3389/fpls.2024.1357316

**Published:** 2024-03-12

**Authors:** Marzia Leporino, Youssef Rouphael, Paolo Bonini, Giuseppe Colla, Mariateresa Cardarelli

**Affiliations:** ^1^ Department of Agriculture and Forest Sciences, University of Tuscia, Viterbo, Italy; ^2^ Department of Agricultural Sciences at the University of Naples, Portici, Italy; ^3^ oloBion SL, Barcelona, Spain; ^4^ Arcadia s.r.l., Rivoli Veronese, Italy

**Keywords:** *Solanum lycopersicum* L., biostimulants, drought, phenotyping, dipeptides, metabolomics

## Abstract

**Introduction:**

High-throughput phenotyping technologies together with metabolomics analysis can speed up the development of highly efficient and effective biostimulants for enhancing crop tolerance to drought stress. The aim of this study was to examine the morphophysiological and metabolic changes in tomato plants foliarly treated with two protein hydrolysates obtained by enzymatic hydrolysis of vegetal proteins from Malvaceae (PH1) or Fabaceae (PH2) in comparison with a control treatment, as well as to investigate the mechanisms involved in the enhancement of plant resistance to repeated drought stress cycles.

**Methods:**

A phenotyping device was used for daily monitoring morphophysiological traits while untargeted metabolomics analysis was carried out in leaves of the best performing treatment based on phenotypic results.

**Results**

PH1 treatment was the most effective in enhancing plant resistance to water stress due to the better recovery of digital biomass and 3D leaf area after each water stress event while PH2 was effective in mitigating water stress only during the recovery period after the first drought stress event. Metabolomics data indicated that PH1 modified primary metabolism by increasing the concentration of dipeptides and fatty acids in comparison with untreated control, as well as secondary metabolism by regulating several compounds like phenols. In contrast, hormones and compounds involved in detoxification or signal molecules against reactive oxygen species were downregulated in comparison with untreated control.

**Conclusion:**

The above findings demonstrated the advantages of a combined phenomics-metabolomics approach for elucidating the relationship between metabolic and morphophysiological changes associated with a biostimulant-mediated increase of crop resistance to repeated water stress events.

## 1 Introduction

Agricultural lands cover approximately 38 percent of the global land surface, and one-third of this is used as cropland (FAO, 2022). This could explain how nowadays crop production and food security may be subject to climate change and how much it will be in the future (Kang et al., 2009; Wang et al., 2023). Climate change refers to changes in climate conditions such as increasing temperatures, changes in precipitation frequency, duration and intensity, and occurring extreme weather events (Skuras & Psaltopoulos, 2012). Climate reports (NOAA, 2023) evidenced a trend from 1880 until now highlighting higher frequency of extreme events especially in the last ten years. One of the outcomes of this unbalanced situation is drought exacerbated by warm and dry conditions with a direct impact on water resources and irrigation requirements (Malek et al., 2018), crop growth conditions, productivity, and cultivation areas. Farmers should find new ways to improve resilience of their crop production aiming at building tolerance against the effects of global warming (Skuras & Psaltopoulos, 2012; Ward, 2022).

In this scenario plant biostimulants could help to preserve crop yields in unfavorable environmental conditions. Among biostimulants, protein hydrolysates (PHs) are gaining a growing interest due to the presence of several beneficial compounds (e.g., peptides, amino acids) that act at multiple levels on plant metabolism increasing nutrient and water use efficiency, yield, and quality, especially when crops grow in unfavorable environmental conditions (Colla et al., 2015; Colla et al., 2017; Van Oosten et al., 2017). Different researchers evidenced the benefits in applying PH on crops subjected to drought stress (Vasconcelos et al., 2009; Colla et al., 2015; Bavaresco et al., 2020; Agliassa et al., 2021). A study performed on tomato grown in open field showed a better water status and pollen viability, and increased yields under drought stress in plants treated with PH in comparison with an untreated control (Francesca et al., 2021). However, in the previous tomato study, PH was applied at high rate (4.8 g/plant) through fertigation system which may have affected plant performance more as nitrogen source than as biostimulant. In another tomato study, Hamedeh et al. (2022) observed in a short tomato trial (8 days) that foliar sprays of a biostimulant based on the complexation of a plant-derived pool of polyphenols with magnesium mitigated the negative effects of water deficit on plants by upregulating genes involved in the carbohydrate metabolism and translocation, stomatal closure, and cell homeostasis, and by stabilizing the levels of the photosynthetic pigments, regulating the accumulation of osmoprotectants, and preserving the cell wall lipid bilayer from oxidation. However, the above tomato study was not able to fully characterize the crop behavior to foliar biostimulant applications over time under multiple drought stress conditions. High-throughput plant phenotyping platforms have been used for studying the biostimulant activity of products through monitoring in a non-destructive way several morpho-physiological plant traits (Rouphael et al., 2018). Moreover, metabolomic analysis of plant tissues has been successfully combined with plant phenotyping to understand the mode of action of plant biostimulants. For instance, Paul et al. (2019) used a phenotyping platform to study the effects of a plant-derived PH on potted tomato plants at early stage of growth under limited water availability, and the best performing treatments were subjected to metabolomic analysis for understanding the mode of action of PH. The phenotyping system was based on multiple stations where plants were moved toward the sensors (‘plant to sensor’) to take information on morpho-physiological traits through RGB, kinetic chlorophyll fluorescence, and thermal imaging. Plant phenotyping revealed that tomato plants grown under low water availability conditions and treated with PH grew better (e.g. higher digital shoot biomass at the end of the trial, and greater relative growth rate over the entire phenotyping period) than control plants. Moreover, metabolomic analysis demonstrated that PH-treated tomato leaves exhibited an improved tolerance to reactive oxygen species in comparison with untreated control leaves. ‘Sensor to plant’ phenotyping platforms have been proposed as alternative to ‘plant to sensor’ platforms for avoiding mechanical disturbance of the plants due to their continuous movements toward the sensors and back to the growing area. A ‘sensor to plant’ phenotyping platform based on a 3D laser scanner implemented by Phenospex (PlantEye F500; Phenospex, Limburg, The Netherlands) was used by Sudiro et al. (2022) to study the effect of two foliar sprays of a plant-based biostimulant on tomato crop recovery after a single water stress event under growth chamber conditions. However, Sudiro et al. (2022) failed to discriminate the treatments effects on morpho-physiological parameters because phenotyping was applied only once at the early beginning of drought stress (4 days after treatment).

Starting from the above considerations, we hypothesized that PHs could improve drought stress recovery of tomato plants to repeated water stress events by modulating morpho-physiological traits and metabolic profile. A greenhouse trial was performed to evaluate the effects of two vegetal-derived PHs on tomato plant recovery after four water stress events. 3D laser scanners (PlantEye F500; Phenospex, Limburg, The Netherlands) were used to monitor daily the morphological traits (digital biomass, 3D leaf area, plant height, plant volume), and plant spectral indices related to the chlorophyll content and plant health. Moreover, metabolic analysis was performed on leaf samples from the best performing PH and control treatment to identify the metabolites differentially expressed on PH-treated leaves.

## 2 Materials and methods

### 2.1 Experiment set up, crop management and growing conditions

The experiment was performed under natural conditions of light and temperature in a polymethyl methacrylate greenhouse at the Experimental Farm of Tuscia University from 20 May 2022 to 22 June 2022 (33 days after transplanting); during the growing cycle the maximum and minimum air temperature was 39 and 16°C, respectively. To reduce excessive temperatures and humidity a ventilation system and side openings were automatically activated when the air temperature exceeded 27°C.

Tomato seeds (*Solanum lycopersicum* L. - cv Syrope F1, Nunhems-BASF, S. Agata Bolognese, Bologna, Italy) were sown in trays (160 holes/tray) filled with a commercial substrate containing mainly peat moss (Brill, Georgsdorf, Germany). The trays were maintained in a growth chamber since the cotyledons reached their full expansion, then they were moved in a greenhouse at 25°C and were regularly watered. After one week the seedlings were fertigated with a solution containing 1 g L^-1^ of a soluble NPK fertilizer (20% N - 8.8% P - 16.6% K), and once they reached two fully expanded leaves, plants were transplanted in 1 litre pots filled with a sandy loam soil (sand 79%, silt 9%, clay 12%), with sub-alcaline pH, medium cation exchange capacity, and low organic matter content. The pots were placed in benches with a plant density of 15 plants/m^2^. The irrigation system was a dripping system with 2 L h^-1^ emitters. During the crop cycle it was used a deltamethrin-based insecticide at the dose of 3 ml L^-1^ to control pests (Decis Evo, Bayer CropScience S.r.l., Milano, Italy).

Before transplanting the water container capacity was measured by saturating three pots and letting them drain for one day before weighing their water holding capacity. During the drain period the surface of the pots was covered with plastic film to avoid evaporation. The water container capacity was calculated as the mean of the weight difference between substrate at water holding capacity and the dry weight of substrate in each pot. Before transplanting all pots were saturated at container capacity and all plants after transplanting were regularly watered for the first week, then the irrigation was cut off to induce water stress. Irrigation was re-applied to saturate the substrate when the plants started showing visible symptoms of water deficit (e.g., beginning of leaf wilting and curling; stem bending), which correspond to a substrate water depletion of 75% of the water holding capacity. Water stress cycles became progressively shorter as air temperature and solar radiation increased, from 9 to 3 days of irrigation interruption.

Fertigation was applied manually two days after transplanting and repeated at mid-cycle with a nutrient solution having an EC of 2.0 dS m^-1^ and a pH of 6.0. The nutrient solution contained the following nutrients: N-NO_3_ (14 mM), P (1m M), S (2.5 m M), K (4 mM), Ca (7 mM), Mg (1.25 mM), Fe (17.1 µM), Mn (17.4 µM), Zn (3.7 µM), B (11 µM), Cu (1.9 µM), Mo (0.5 µM). Seventy-two plants were distributed according to a randomized complete block design with 12 replicates. Each replicate was composed by one plant per treatment. The following treatments were tested during the water stress cycles: two prototypes of plant-derived protein hydrolysates (PH1 and PH2) and a control treatment. PH1 and PH2 were obtained through enzymatic hydrolysis in the biostimulant discovery platform of Hello Nature USA (Anderson, IN 46016, US). PHs were applied as foliar spray diluted at the dose of 3 ml L^-1^ and control treatment sprayed with distilled water was also included. PH1 was a Malvaceae-derived PH containing 16.9% carbon, and 4.67% nitrogen as free aminoacids and peptides. The aminogram has been reported by El-Nakhel et al. (2023). PH2 was a Fabaceae-derived PH having a carbon content of 20.0%, and 4.9% of nitrogen as free aminoacids and soluble peptides. The PH2 composition has been reported by Ceccarelli et al. (2021). PHs were applied weekly for a total of 5 treatments starting 3 days after transplanting until 2 days before the end of the trial, and they were applied in the late afternoon when the conditions were more favorable (lower temperature and higher humidity) for leaf absorption. The solution was sprayed with a 2 L manual sprayer (Volpitech 2; Volpi, Casalromano, Italy) until it was running off leaves. During each foliar spraying event, pot surfaces were covered with plastic films to avoid substrate contamination with biostimulant treatments.

### 2.2 Plant phenotyping

**Figure 1:**
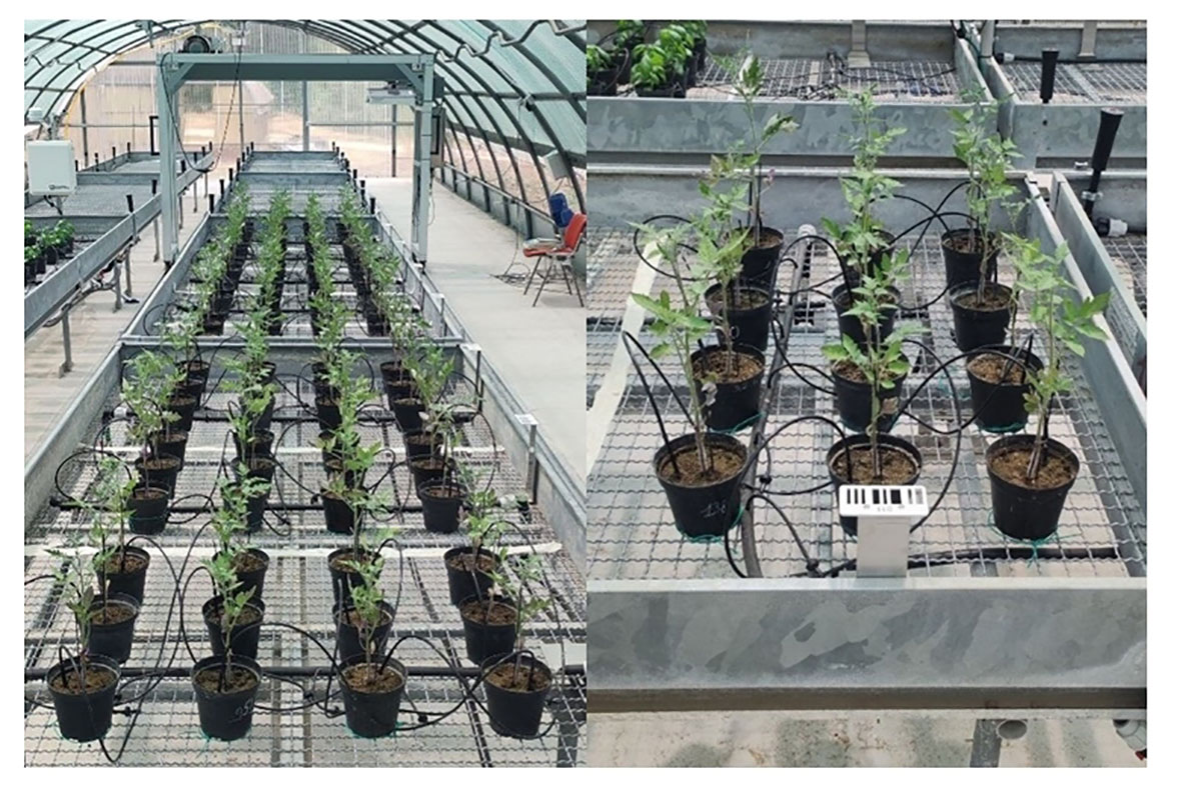
Experimental plots placed on the benches in correspondence of the barcodes detected by the PlantEye F500 - Multispectral 3D laser scanners.

A high-throughput phenotyping platform, belonging to Arcadia Spin-off Company (Rivoli Veronese, Italy), located at the Experimental Farm of Tuscia University was used to monitor morpho-physiological parameters of tomato plants. The platform consisted of two sensors (PlantEye F500 - Multispectral 3D laser scanners; Phenospex, Herleen, The Netherlands) able to detect a cloud of points/pixels corresponding to the plants shape. Simultaneously, a flashing light lit the plants and measured in different wavelengths to acquire information about the state of health. According to the data acquisition method, the plots were recognized by the sensors through barcodes (**Figure 1**).

The experimental design was set up on a software connected to the platform to monitor morpho-physiological plant traits (HortControl; Phenospex, Herleen, The Netherlands). Plants were scanned daily for the whole crop cycle. The scans were carried out in the central hours of the day when the atmospheric evaporative demand and the water stress effects on plants were highest.

Three main morphological crop traits were explored with the multispectral 3D laser scanners: digital biomass, 3D leaf area, and plant height. These traits are related to each other because digital biomass is the product of the leaf area and plant height. 3D leaf area is also linked to the projected leaf area, that is a parameter calculated on the section plane corresponding to the height of the pot set manually on the software. The following physiological parameters were explored thanks to the visible light and infrared spectra: Normalized Difference Vegetation Index (NDVI), Normalized Pigment Chlorophyll ratio Index (NPCI), Plant Senescence Reflectance Index (PSRI), Green Leaf Index (GLI), and Hue.

NDVI is widely used to study the plant photosynthetic efficiency and it is calculated as follow:


$$\frac{NIR\;–\;RED}{NIR\;+\;RED}$$

where the peak wavelength for NIR and RED was 720-750nm and 620-645nm, respectively.

NPCI is used to estimate chlorophyll content through the following formula:


$$\frac{RED-BLUE}{RED+BLUE}$$

where BLUE had a peak wavelength of 460-485nm.

NPCI values decrease when the chlorophyll content is higher.

PSRI represents the leaf senescence, and it is the ratio of carotenoids and chlorophyll detected:


$$\frac{RED-GREEN}{NIR}$$

where green had a peak wavelength of 530-540nm.

PSRI curve follows the plant development, so it has high values at seedling stage when plants are small and the chlorophyll content is concentrated in a small amount of tissue, lower values when the plant grows and the chlorophyll values predominate the carotenoids, and again an increasing trend during leaf senescence.

GLI quantifies the green parts of the plants as follows:


$$\frac{2*\left(GREEN-\;RED-BLUE\right)}{2*\left(GREEN\;+\;RED+BLUE\right)}$$

It represents the relation between the reflectance in the green channel compared to the other two visible light channels (red and blue), so the higher the green reflection compared to the other channels the higher the green leaf index.

These parameters are expressed in a range between – 1 and 1, except the Hue that is represented as an angle between 0° and 360°, allowing to classify a color as red, yellow, green, blue, or an intermediate between any contiguous pair of these colors.

NDVI, NPCI, PSRI, GLI, and Hue can be determined as average values or as bins representing the ranges in which plant pixels can fall depending on plant health. Four ranges for each index were generated for deeply explore the changes in spectral indexes.

### 2.3 Metabolomics analysis

At the end of trial (22/06/2022; 33 days after transplanting), when the tomato plants have been subjected to 5 foliar biostimulant treatments and 4 water stress cycles, 3 young leaves (third leaf from the growing tip) per plot were harvested and immediately frozen with liquid nitrogen and stored at -20°C. The 3 leaves from each replicate were pooled and homogenized, then metabolites were extracted in acidified 80% methanol, as previously reported by Paul et al., 2019. The samples were extracted by Ultra- Turrax (Ika T-25; Staufen, Germany), centrifuged and filtered through a 0.22 µm cellulose membrane into vials for analysis. Untargeted metabolomic analysis and metabolite identification in samples was performed at oloBion Laboratory (Barcelona, Spain) following the procedure described by Bonini et al. (2020).

### 2.4 Statistical analysis

The scan process may produce some noises due to the presence of pixels that correspond to the barcode, the benches or elements that may interfere with data detection. The software (HortControl, Phenospex, Heerlen, The Netherlands) allowed data reprocessing to remove the errors, then the dataset was downloaded and prepared for the statistical and graphical analysis using RStudio (RStudio Team, Vienna, Austria). First, the Pearson correlation coefficients were calculated and reunited in a correlation matrix including all variables derived from the phenotyping platform. Then, a correlation plot was created to observe the relationship between morphological and physiological parameters. For each treatment, linear regression analysis expressed as y = ax +b (where a is the slope and b is the y-intercept), was performed for Digital Biomass during each recovery period to evaluate the recovery rates (slopes of linear regression models) of stressed plant following re-irrigation. The effect of vegetal-protein hydrolysates was analyzed on Digital Biomass, 3D Leaf Area and Plant Height by one-way ANOVA and *post-hoc* test was Tukey’s test (p= 0.05). For each treatment the mean and standard error were calculated using the values of 12 plants. Metabolomic data was processed by oloMAP 2.0^2^ created in oloBion laboratory (Bonini et al., 2020).

## 3 Results

### 3.1 Morphophysiological traits and their correlations

**Figure 2:**
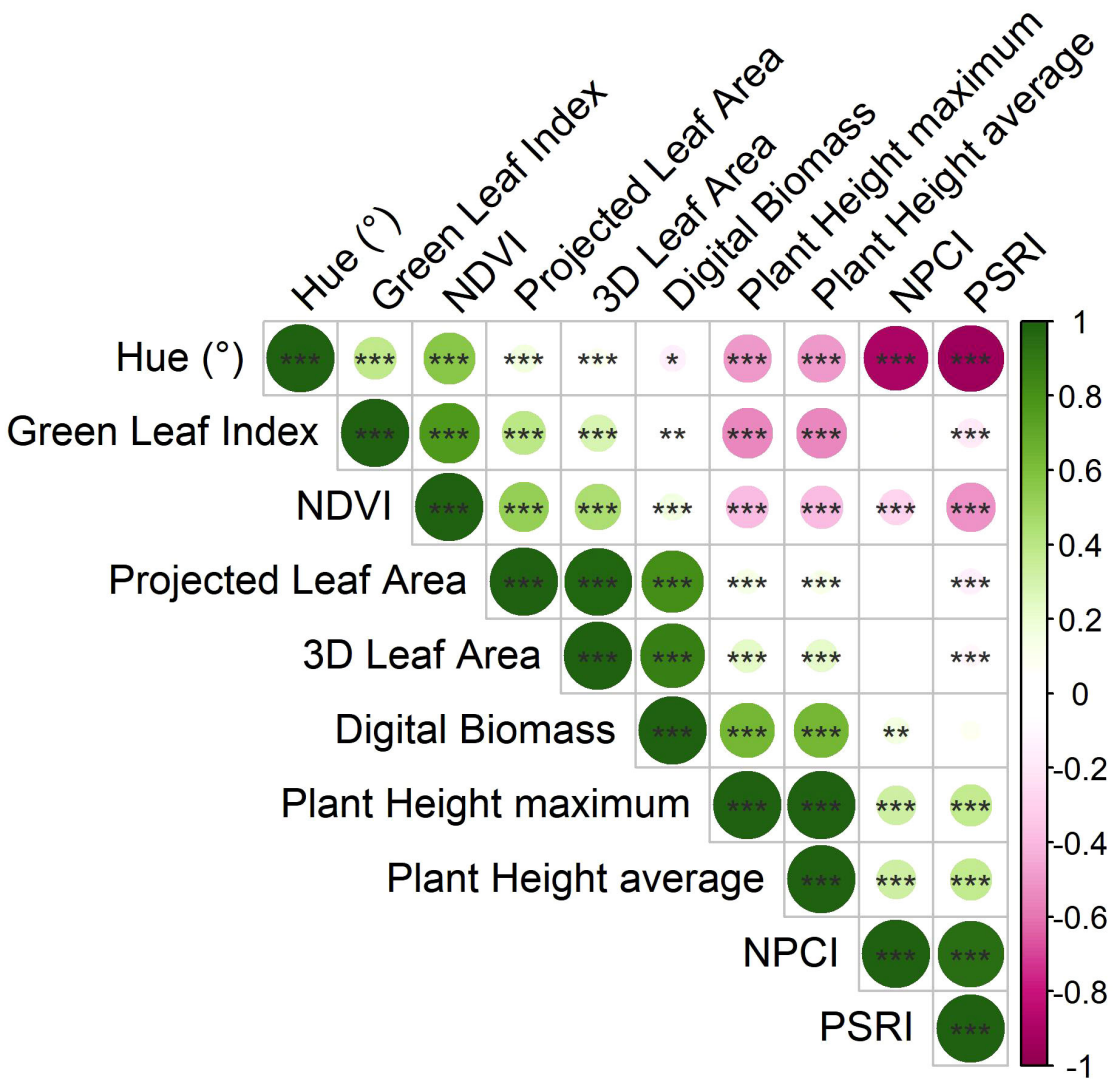
Correlation plot derived from Pearson’s correlation coefficients computed on the morpho-physiological traits calculated by the HortControl software. Positive correlations, having correlation coefficients from 0 to 1, are represented by green colors, while negative correlations, having correlation coefficients from 0 to -1, are in pink. *, **, and *** are significant at p < 0.05, 0.01 and 0.001, respectively (t-test between pairs of variables).

Pearson’s correlation coefficients were computed to investigate the relationships among morphological and physiological traits resulting from imaging phenotypes (**Figure 2**).

Digital biomass was significantly positively correlated to 3D leaf area (r = 0.880***) and the plant height (r = 0.630***). Moreover, there was a significant positive correlation between 3D leaf area and projected leaf area (r = 0.979***), and plant height average and plant height maximum (r = 0.999***), respectively. Among the physiological traits, there was a significant positive correlation between Normalized Pigments Chlorophyll ratio index (NPCI) and Plant Senescence Reflectance Index (PSRI) (r = 0.945***), while they were both negatively correlated to Normalized Digital Vegetation Index (NDVI) (r = -0.271***, and r = -0.517***, respectively), and Hue (r = -0.890***, and r = -0.942***, respectively). NDVI was positively correlated to Hue (r = 0.564***), and Green Leaf Index (GLI) (r = 0.776***). Concerning the relationship between morphological and physiological parameters, a negative correlation between plant height average and Hue (r = -0.491***), GLI (r = -0.541***), and NDVI (-0.388***) was recorded.

**Figure 3:**
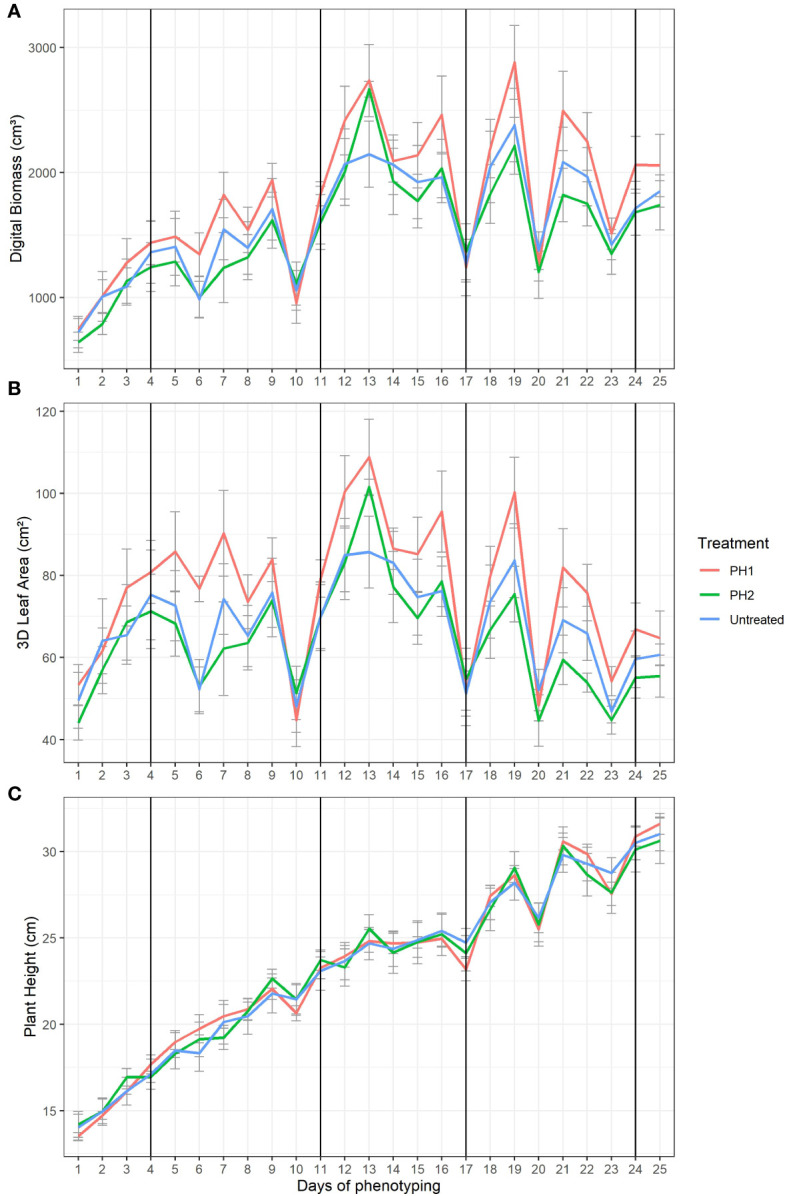
Digital Biomass **(A)**, 3D Leaf Area **(B)**, Plant Height **(C)** from HortControl software during the trial. The x axis represents the days in which the scans were made, and the black vertical lines correspond to the days when the biostimulant treatments were applied. PH1 and PH2 represent a Malvaceae- and the Fabaceae-derived protein hydrolysate, respectively. Bars indicate standard errors of the means.

The effect of the two PHs in mitigating drought stress of tomato plants was explored on digital biomass (**Figure 3A**), 3D leaf area (**Figure 3B**), and plant height (**Figure 3C**). Four major water stress events (10, 17, 20 and 23 days) were evident in the **Figure 3** where digital biomass, 3D leaf area and plant height strongly decreased. The drop of digital biomass, 3D leaf area and plant height values was related to leaf wilting/curling and stem bending. After irrigation, plants needed 3 (from 10 to 13 day), 2 (from 17 to19 day), 1 (from 20 to 21 day), and 1 (from 23 to 24 day) days after water stress to recover (restore turgor of stems and leaves) in the first, second, third, and fourth stress event, respectively. Tomato plants treated with PH1 reached the highest values for digital biomass and 3D leaf area at the end of each recovery period (**Figures 3A, B**) in comparison to control treatment. In the first water stress event, PH2 performed similarly to PH1 while in the following stress events the performances of PH2 were similar to control treatment.

**Figure 4:**
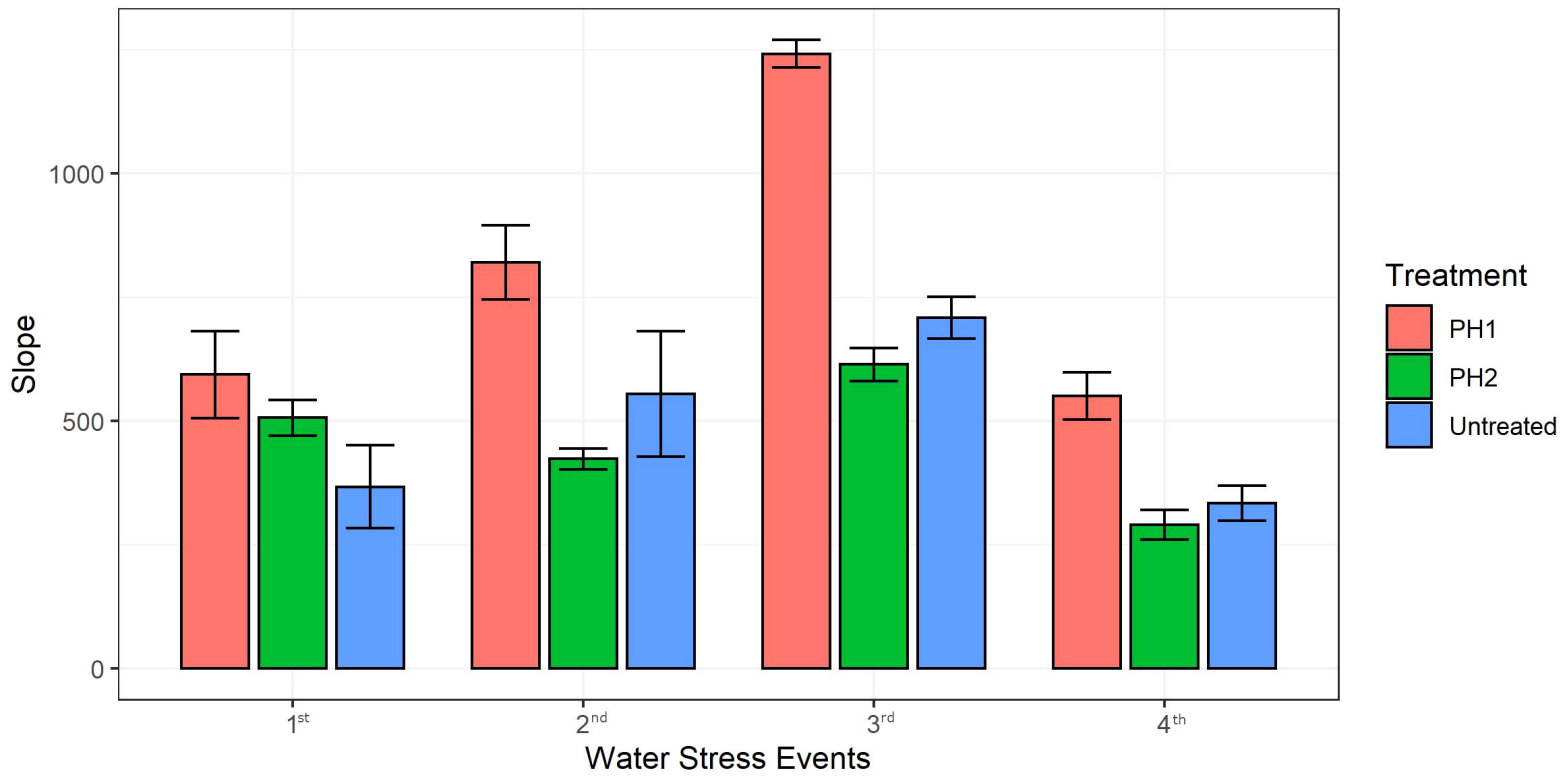
Slopes of the linear regression models (y = ax +b where a is the slope and b is the y-intercept) describing the relationship between Digital Biomass (y) and days (x) of the recovery period upon re-irrigation after each water stress event (1^st^, 2^nd^, 3^rd^, 4^th^). PH1 and PH2 represent a Malvaceae- or Fabaceae-derived protein hydrolysate, respectively. Bars indicate standard errors of the means.

Linear regression analysis was performed to predict recovery rate (slopes of the linear models) of digital biomass in all treatments after each water stress event upon re-irrigation (**Figure 4**). Coefficients of determination (R^2^) were always higher than 0.91 for all linear regression models (data not shown). The slopes of linear regression for PH1 were significantly higher by 62, 48, 75 and 65% than untreated control during the recovery period after the first, second, third and fourth stress event, respectively (**Figure 4**). Except for the first stress event where the slope of PH2 linear regression during the recovery period was significantly higher than that of control treatment (506.5 vs 367.4), the slopes of PH2 linear regressions were similar to those of the control treatment after the second, third, and fourth stress event (**Figure 4**).

**Figure 5:**
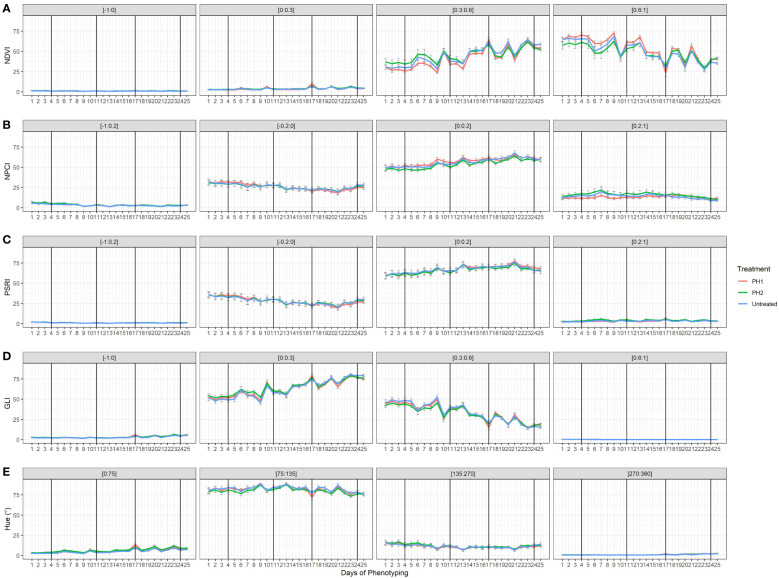
Plant spectral indices [Normalized Digital Vegetation Index (NDVI; **(A)**, Normalized Pigments Chlorophyll ratio index (NPCI; **(B)**, Plant Senescence Reflectance Index (PSRI; **(C)**, Green Leaf Index (GLI; **(D)**, HUE average (Hue; **(E)**] from HortControl software during the trial. The x axis represents the days in which the scans were made, and the black vertical lines correspond to the days when the biostimulant treatments were applied. For each plant spectral index, the pixels values that fall within the ranges of numbers reported above each chart are displayed. PH1 and PH2 represent a Malvaceae- and the Fabaceae-derived protein hydrolysate, respectively. Bars represent standard errors of the means.

The effect of the two PHs on NDVI, NPCI, PSRI, GLI, and Hue was also evaluated during the trial (**Figure 5**). Concerning NDVI, plant pixels were mainly grouped in the [0.3:0.6] and [0.6:1] ranges. Untreated and PH2-treated plants showed higher percentages in the lower range [0.3:0.6], while PH1 had higher percentages in the [0.6:1] range. Plant pixels of GLI were mainly in the [0:0.3] and [0.3:0.6] ranges with similar values among treatments. In all treatments, NDVI and GLI values dropped at each stress event and then rose again during the recovery periods (re-irrigation). Moreover, NDVI and GLI declined over the growing cycle in the [0.6:1] and [0.3:0.6] ranges, respectively. On the contrary, NDVI and GLI increased over the growing cycle in the [0.3:0.6]: and [0:0.3] ranges, respectively. In all treatments, NPCI and PSRI values were mainly concentrated in the [0:0.2] range, with an increase of the values over time whereas NPCI and PSRI of the [-0.2:0] range declined over time (**Figure 5**). Hue (°) showed most of the plant pixels grouped in the [75:135] range interval with similar behavior among treatments.

### 3.2 Untargeted metabolomic analysis

**Figure 6:**
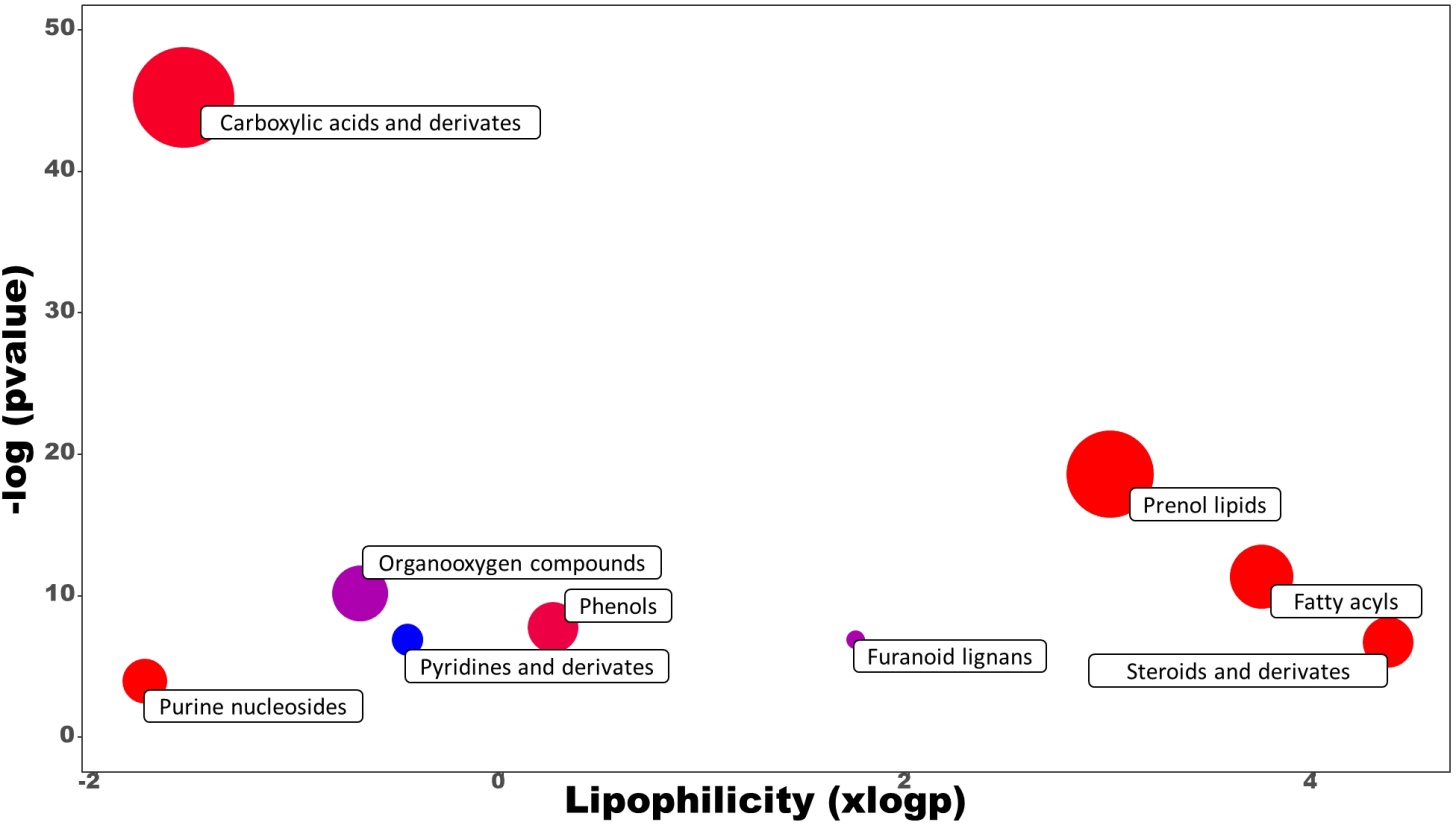
Chemical enrichment analysis (ChemRICH) of statistically different annotated metabolites in Malvaceae-derived protein hydrolysate (PH1) treated leaves compared to control treatment at the end of the trial. Upregulated or downregulated clusters are indicated by different colors as follows: red = up-modulation, blue = down-modulation, purple = up-/down-modulation.

Untargeted metabolomics was performed on leaves sampled at the last day of the trial to investigate the mode of action of the best performing PH treatment (PH1) in comparison with control treatment. This approach allowed to explore the entire metabolome influenced by the PH1 treatment. 191 metabolites were identified, 54 of them were significantly modulated by the PH1. Compounds subjected to chemical enrichment analysis with ChemRICH were grouped into clusters, highlighting metabolites belonging both to the primary and secondary metabolism (**Figure 6**).

Carboxylic acids and prenol lipids were the largest classes affected, in which most of the metabolites identified were upregulated in the PH1 treatment in comparison to the control one. Among the carboxylic acids, PH1 applications promoted the accumulation of dipeptides, such as Leu-Phe (with the highest increase corresponding to a fold change of 14.8 x), PyroGlu-Val (9.7 x), Arg-Phe (8.7 x), Leu-Leu (8.5 x) followed by many other dipeptides (Arg-Leu, Val-Pro, Val-Leu, Glu-Phe, Gly-Leu, Asn-Leu) that also showed an increase compared to control treatment. Conversely, metabolic intermediates such as (R)-S-lactoylglutathione and S-formylglutathione were downregulated in comparison with control treatment (0.73 x and 0.67 x respectively). Also, the pathway related to hormones biosynthesis was negatively modulated as demonstrated by the increase of methylated forms of gibberellins (A4 with a fold change of 1.50 x, and A34 1.61 x) within the prenol lipids, and 5-methyl-DL-tryptophan (1.95 x) within the indoles. Another compound that was downexpressed was scopolin, a glucoside of scopoletin (0.67 x), while interesting classes such as fatty acids and phenols were upmodulated (with average fold change of 1.48 x and 1.46 x, respectively).

## 4 Discussion

In this study, omics technologies such as high-throughput plant phenotyping and metabolomics analysis were used to evaluate the effects of two plant-derived protein hydrolysates (PHs) on relieving drought stress on tomato plants. Phenospex platform was primarily used in plant phenotyping to validate the instrument performing phenotypic measurements (Kjaer and Ottosen, 2015; Manavalan et al., 2021) or to study the crop response to stress such as wheat under salt stress (Lancelot et al., 2016) and okra under flooding stress (Schafleitner et al., 2021). In the current trial, the first goal was to investigate the relationship between the most important morpho-physiological traits recorded by the two multispectral 3D laser scanners. Digital biomass increased during growing cycle, and it had a strong positive correlation with its components (3D leaf area and plant height). Digital biomass was mainly correlated to 3D leaf area (r = 0.880***) than plant height (r=0.630***), as reported by Laxman et al. (2018) who examined digital biomass and predicted leaf area, linking both traits to fresh biomass. A strong positive correlation was also found between 3D leaf area and the projected leaf area on the section plane defined on the software (matching to the pot height). Furthermore, the maximum height (the highest pixel recorded by the scanner) and the average height (recorded as the average of the point cloud values of the plants) were positively correlated. Even though both traits had nearly identical values, the average height might be used in cases where plants develop irregularly, subjecting the maximum height to greater measurement error. Considering the spectral indices, the average NDVI was found to be negatively correlated with both the average NPCI and the average PSRI. NDVI has been described as one of the most important vegetative indices as well as a stress detector for revealing health status of the plants (Katsoulas et al., 2016). In all treatments NDVI dropped in the [0.6:1] range which corresponds to very healthy plants (**Figure 5**); NDVI of [0.6:1] range decreased over time due to the multiple stress events with highest values for PH1 treatments in most scan days. The better healthy status of plants in PH1 treatments was also confirmed by the lower pixels of PH1 treated plants in the NDVI range [0.3:0.6] in comparison with PH2 and untreated plants for most of the scan days. NPCI and PSRI behaved similarly throughout the growth cycle, suggesting an increase in chlorophyll content in leaf tissues (which supports the NDVI results). In contrast to NDVI, which showed an important drop when the stress was imposed, NPCI and PSRI were less influenced by water stress while they are usually more affected in the senescence phases of plants (Merzlyak et al., 1999; Hatfield and Prueger, 2010). NDVI was also positively correlated to Hue and GLI because Hue values were concentrated mostly in the green range [75:135] (Yang et al., 2015), and GLI values were mainly in ranges corresponding to higher reflectance in the green band (Louhaichi et al., 2001).

Except for the first water stress event, where the PH2 behaved similarly to the PH1, plants treated with PH1 always displayed the highest ability to recover from the drought stress events (**Figure 3**). The results are in line with a previous study where the application of a biostimulant containing vitamins, amino acids, proteins, and betaines, improved tomato tolerance to drought stress (Petrozza et al., 2014). The beneficial effect of PH1 was more evident in the 3D leaf area where the PH1 provided generally higher 3D leaf area compared to PH2 and control treatment (**Figure 3**). Plant height was less sensitive to water stress and PH application than 3D leaf area indicating that 3D leaf area is a more suitable morphological trait for screening plant biostimulants under water stress conditions.

The slopes of the linear regression models, describing the relationship between digital biomass and days of the recovery period after each water stress event for the three treatments, were determined for evaluating the drought-stress recovery effect of various biostimulant treatments. The results highlighted that Malvaceae-derived PH (PH1) was highly effective in enhancing plant recovery after all drought stress events while the positive effect of Fabaceae-derived PH (PH2) was detected only during the recovery period after the first stress event. The above findings are in line with a study of Domingo et al. (2023) where foliar applications of a vegetal protein hydrolysate conferred drought resistance to tomato plants resulting in higher biomass and shoot height.

Metabolomic results allowed to investigate the changes induced by the best performing PH treatment (PH1) on metabolic profile of leaves. Among carboxylic acids, dipeptides were the most prevalent compounds stimulated by the PH1, and most of them had larger fold changes than other metabolites (**Table 1**). Dipeptides represent an emerging class of small molecules playing a pivotal role in nitrogen distribution and as regulators of plant metabolism, including response to abiotic stress (Minen et al., 2023). The biogenesis of these molecules was identified mainly from protein degradation in plant cells, and especially in the activation of autophagy mechanisms (Calderan-Rodrigues et al., 2021). Recently, it was discovered in *Arabidopsis* that dipeptides accumulated in leaves under heat stress and dark conditions, and the accumulation was more pronounced for Asp- and Glu-containing dipeptides (Thirumalaikumar et al., 2020). Moreno et al. (2021) reported that Tyr-Asp enhanced growth of both Arabidopsis and tobacco plants under oxidative stress conditions, by inhibiting the activity of a key glycolytic enzyme and redirecting glycolytic triose-phosphates toward the pentose phosphate pathway and NADPH production. Similarly to the previous works, Calderan-Rodrigues et al. (2021), discovered that proteogenic dipeptides operate as metabolic switches, regulating essential enzyme activities involved in carbon flow distribution. Dipeptides were shown to be more abundant in short-day diel cycles, particularly dipeptides containing glucogenic amino acids like Pro, Asp, Ser, and Val, suggesting the possibility that they might act as alternate respiratory substrates to support plant growth. Since plant metabolism and growth are sustained by carbon reserves mobilization during the dark period, dipeptides composed by glucogenic amino acids (Asp, Arg, Glu, Gln, Gly, His, Met, Ser, Val), can represent a source for the genesis of carbon skeletons to produce pyruvate and then glucose through gluconeogenesis (Eastmond et al., 2015) to support loss of carbon reserves. In the current trial, drought stress conditions may have caused carbohydrate starvation due to the reduction of photosynthesis rates and the increase of sugar production as osmolytes. In such conditions, tomato leaves treated with PH1 showed a strong increase of dipeptides containing glucogenic amino acids (Arg-Leu, Arg-Phe, Asp-Leu, Asp-Phe, Glu-Phe, Gly-Leu, PyroGlu-Val, Thr-Val, Val-Leu, Val-Pro) in comparison with control leaves (**Table 1**); dipeptides containing glucogenic amino acids could have mitigated water stress on plants by acting as carbon source for plant growth under photosynthesis-limiting conditions (drought stress events). Moreover, drought stress has been associated to oxidative damage due to the over production of reactive oxygen species (Samaranayaka and Li-Chan, 2011). In contrast to the increased dipeptides, several compounds involved in detoxification of byproducts of plant primary metabolism, or in plant defense against (a)biotic stresses, were down-accumulated in PH1-treated leaves in comparison with control leaves. Plants treated with PH1 had decreased levels of (R)-S-lactoylglutathione, which is involved in the breakdown of methylglyoxal, a cytotoxic consequence of glycolysis (Ghosh et al., 2014). The similar fate occurred S-formylglutathione, which is implicated in formaldehyde detoxification (Haslam et al., 2002). Scopolin (a coumarin derivative of a specific pathway of the phenylalanine), having a role as signal molecule against reactive oxygen species (Döll et al., 2018), also decreased. Because certain dipeptides, such as those containing hydrophobic amino acid residues like Val or Leu at the N-terminus, were shown to have reactive oxygen-scavenging properties (Sultana et al., 2022), it is reasonable to assume that dipeptide accumulation in PH1-treated leaves could have contributed to mitigate the oxidative damage induced by water stress. The increase of dipeptides in PH1-treated leaves could have been determined by the exogenous supply of dipeptides from the PH1 or by enhancing the accumulation of endogenously produced dipeptides in plant cells. The downmodulation of detoxifying compounds in PH1-treated leaves can be linked not only to the increase of dipeptides but also to the enhance of other compounds having antioxidant properties like phenols (Kumar et al., 2020), and with the modulation of fatty acids (Zhong et al., 2011; Ullah et al., 2022) such as the unsaturated omega-3 fatty acids stearidonic acid.

**Table 1:** Effects of the PH1 on the variation of compounds (up or down-regulation) in tomato leaves in comparison with control treatment.

Cluster	Compound name	p value	Fold change	up/Down
5’-deoxyribonucleosides	S-adenosyl-1,8-diamino-3-thiooctane	0.000	35.064	up
Carboxylic acids and derivatives	(3R)-beta-phenylalanine	0.004	1.684	up
	(R)-S-lactoylglutathione	0.004	0.733	down
	Ala-Trp	0.032	1.681	up
	Arg-Leu	0.000	5.839	up
	Arg-Phe	0.000	8.708	up
	Asn-Leu	0.000	3.046	up
	Asp-Leu	0.035	1.629	up
	Asp-Phe	0.029	1.316	up
	Captopril	0.000	3.846	up
	Glu-Phe	0.000	3.323	up
	Gly-Leu	0.000	3.188	up
	L-4-hydroxyglutamate semialdehyde	0.008	1.392	up
	L-Arginine	0.000	2.492	up
	Leu-Leu	0.012	8.548	up
	Leu-Phe	0.011	14.852	up
	Leu-Pro	0.002	2.026	up
	Leu-Trp	0.038	1.572	up
	L-nicotianamine	0.022	1.709	up
	L-Tyrosine	0.008	1.424	up
	N-Acetyl-D-phenylalanine	0.000	5.815	up
	pantetheine	0.000	2.780	up
	PyroGlu-Val	0.000	9.700	up
	S-formylglutathione	0.012	0.671	down
	Thr-Leu	0.001	2.051	up
	Thr-Val	0.012	1.882	up
	Tyr-Ile	0.045	1.637	up
	Val-Leu	0.027	4.269	up
	Val-Pro	0.000	4.725	up
	valylphenylalanine	0.000	2.656	up
Coumarins and derivatives	scopolin	0.010	0.673	down
Fatty Acyls	(Z)-6,9,10-Trihydroxyoctadec-7-enoic acid	0.023	1.359	up
	9-Oxo-10(E),12(E)-octadecadienoic acid	0.009	1.684	up
	chromomoric acid B	0.026	1.411	up
	stearidonic acid	0.043	1.406	up
Furanoid lignans	(-)-yatein	0.045	1.486	up
Imidazopyrimidines	2-dimethylamino-6-hydroxypurine	0.045	0.748	down
	isoguanine	0.029	1.874	up
Indoles and derivatives	5-methyl-DL-tryptophan	0.010	1.951	up
Organonitrogen compounds	2-amino-1-phenylethanol	0.000	2.820	up
	2-phenylethyl beta-D-glucopyranoside	0.010	3.804	up
	salicin	0.006	0.700	down
Phenols	capsiconiate	0.047	1.321	up
	dimeric urushiol peroxide	0.028	1.555	up
	DL-Octopamine	0.002	1.519	up
Prenol lipids	16,17-dihydro-16alpha,17-dihydroxy gibberellin A12	0.031	1.550	up
	2alpha, 7beta-dihydroxytaxusin	0.033	1.407	up
	alpha-Cyperone	0.027	1.264	up
	methyl gibberellin A34	0.026	1.609	up
	methyl gibberellin A4	0.021	1.500	up
	oleanolate 3 beta-D-glucuronoside	0.015	1.380	up
Pyridines and derivatives	pyridoxal	0.046	0.787	down
Steroids and steroid derivatives	beta1-tomatine	0.006	1.133	up
Tetrahydroisoquinolines	1,2,3,4-tetrahydro-6,7-isoquinolinediol	0.038	1.271	up

Significant p values (<0.05) are also displayed.

Moreover, Luziatelli et al. (2019) observed that biostimulant effects of the foliar applications of a commercial vegetal derived PH on lettuce was associated to changes of microbial community in the phyllosphere with an enhancement of plant growth promoting bacteria population. Therefore, a microbial-mediated improvement of drought stress resistance in PH1-treated tomato plants cannot be excluded.

The inactive gibberellic acid form (Methyl gibberellins) increased in leaves of plants treated with PH1 in comparison with control treatment. Nir et al. (2013) observed higher tolerance to drought in transgenic tomato with reduced levels of active forms of gibberellins, probably counteracting simultaneously the action of 5-methyl-DL-tryptophan that conversely represents a growth inhibitor (Yamada et al., 2009). This was the case in the current trial where the increase of inactive gibberellin forms in PH1-treated leaves was associate with an improvement of drought resistance in tomato plants.

## 5 Conclusion

Today there is an urgent need to improve agricultural resilience against increasingly frequent extreme events such as drought. Plant biostimulants such as protein hydrolysates (PHs) could represent a useful tool to mitigate the negative effects of drought on tomato plants. In the current experiment, a novel approach combining high throughput plant phenotyping and metabolomics was employed to investigate the influence of two foliarly applied PHs (Malvaceae (PH1) or Fabaceae (PH2)-derived PH) on drought stress recovery upon reirrigation in tomato plants. The phenotyping results on digital biomass showed that PH1 was more effective for promoting plant recovery after multiple water stress events than PH2. The observed variations of digital biomass during multiple stress events were mainly linked to changes on 3D leaf area indicating that 3D leaf area can also be considered a suitable phenotypic marker for screening biostimulants for drought stress mitigation. Metabolomic analysis of leaves indicated that PH1-mediated improvement of drought stress resistance in tomato plants was mainly associated with an increase of dipeptides, a class of small compounds that are gaining interest due to their properties in enhancing plant tolerance to (a)biotic stress. Further studies are needed to better understand if the observed dipeptide changes in leaf tissues were caused by the direct supply of peptides from PH or by the stimulation of endogenous peptide accumulation.

## Data availability statement

The raw data supporting the conclusions of this article will be made available by the authors, without undue reservation.

## Author contributions

ML: Data curation, Formal analysis, Investigation, Methodology, Software, Validation, Visualization, Writing – original draft, Writing – review & editing. YR: Writing – review & editing. PB: Conceptualization, Data curation, Formal analysis, Investigation, Methodology, Project administration, Software, Supervision, Validation, Visualization, Writing – review & editing. GC: Conceptualization, Data curation, Formal analysis, Funding acquisition, Investigation, Methodology, Project administration, Resources, Software, Supervision, Validation, Visualization, Writing – original draft, Writing – review & editing. MC: Conceptualization, Data curation, Formal analysis, Methodology, Software, Validation, Visualization, Writing – original draft, Writing – review & editing.
